# The effects of digitalisation on health and social care work: a qualitative descriptive study of the perceptions of professionals and managers

**DOI:** 10.1186/s12913-023-09730-y

**Published:** 2023-06-30

**Authors:** Anu-Marja Kaihlanen, Elina Laukka, Janna Nadav, Johanna Närvänen, Petra Saukkonen, Juha Koivisto, Tarja Heponiemi

**Affiliations:** 1 Finnish Institute for Health and Welfare, PO Box 30, 00271 Helsinki, Finland; 2 Research Unit of Nursing Science and Health Management, University of Oulu, 90230 Oulu, Finland; 3 Department of Health and Social Management, University of Eastern Finland, Kuopio, Finland

**Keywords:** Health and social care professionals, Digitalisation of work, Digital technology, Digital services implementation, Digital health, Changing work, Managers, Interview, Focus groups

## Abstract

**Background:**

Today, digitalisation is strongly present in health and social care, and it increasingly affects the organisation of work, work requirements, tasks and tools. Due to the constant change in work, up-to-date knowledge is needed about these micro-level effects of digitalisation and how professionals experience the effects in their work.

Furthermore, even though managers play a key role in implementing new digital services, their perceptions of the effects of digitalisation and whether they match the views of professionals remain unknown. This study examined how health and social care professionals and managers perceive the effects of digitalisation on the work of professionals.

**Methods:**

We used a qualitative approach and conducted eight semi-structured focus group interviews with health and social care professionals (*n* = 30) and 21 individual interviews with managers in 2020 in four health centres in Finland. The qualitative content analysis included both an inductive and a deductive approach.

**Results:**

Digitalisation was perceived to have changed professionals’ 1) workload and pace, 2) the field and nature of work, 3) work community communication and interaction, and 4) information flow and security. Both professionals and managers identified effects such as accelerated work, reduction in workload, constant learning of technical skills, complicated work due to vulnerable information systems, and reduction in face-to-face encounters. However, managers did not bring up all the effects that professionals considered important, such as the creation of new work tasks, increased and duplicated work, or insufficient time to get acquainted with the systems.

**Conclusions:**

The findings suggest that some of the effects of digitalisation on professionals’ work and changes in the workplace may receive too little or no recognition from managers. This increases the risk that the potential negative effects may be overlooked and that managers will adopt systems that do not support the work of professionals. To reach a common understanding of the effects of digitalisation, continuous discussions between employees and different management levels are required. This contributes to professionals’ well-being and adaptation to changes, as well as the provision of quality health and social services.

## Background

Digitalisation has a robust presence today in many health and social care functions. With the COVID-19 pandemic, the increased need for digital services has been evident and has required health and social care systems and professionals to rapidly expand more services into a digital format to reduce contact and prevent infections [1, 2]. The range of digital services, information systems and platforms increased significantly even before the pandemic, and simultaneously affected the daily work of health and social care professionals [3, 4].

Digitalisation requires changes in both the work and the work culture of organisations and units. However, changing established ways of working can be challenging and professionals may experience difficulties in adapting new digital technologies to work practices [3]. Global concerns have been raised about the preparedness, capacity and adaptation of professionals to this fairly rapid digital transformation of work [5, 6]. An up-to-date understanding of the experiences of health and social care professionals regarding increased digitalisation and its effects on their work is important, as they play a key role in achieving the goals of digital transformation, such as improving productivity, efficiency, information flow and quality of care [7, 8].

To achieve the intended positive outcomes and the successful adoption and integration of various digital services into professionals’ daily work, the literature emphasises the need for co-design and involving professionals in decision-making regarding their implementation [9–12]. However, to find ways to promote the benefits of digitalisation and eliminate the potential risks of it, it is crucial that managers also have appropriate knowledge of how employees perceive digitalisation and its impact on their work [13]. Managers should identify the needs of their employees in a digital work environment to adapt their management behaviour to them [14]. The role of managers is crucial in supporting the adaptation, the development of skills and the optimisation of the workflows of professionals when implementing and using new digital services [15, 16]. Moreover, managers, especially at the senior management level, are responsible for making the decision to implement new digital solutions [17]. Thus, it is essential for them to understand the effects of implementations on professionals’ workflow and what the potential benefits and hindrances are [18, 19].

Digitalisation of work is known to lead to changes in professionals’ work tasks, tools and work organisation, while significantly increasing the work requirements, such as developing the skills needed to adapt to digital transformation [20, 21]. The digital transformation of work can be defined as a phenomenon in which new technologies cause significant changes in many aspects: how employees perform tasks and processes, their social relationships within and outside the organisation, and subsequently their overall work experience [22]. Digital transformation has been widely scrutinised at the macro level, for example, to assess its economic and societal impacts [23, 24]. Less attention has been paid to mid-level impacts, such as changes in the processes and structures of organisations, and even less to micro-level impacts, meaning the impact and changes that digitalisation brings to an individual’s work and work environment [22]. The latter has often been focused on professionals’ experiences of different digital services [25–27] and their usability [28, 29], because according to the technology acceptance model (TAM), the successful utilisation of technologies at the user level can be predicted based on their perceived ease of use and usefulness [30, 31].

Digitalisation has been expected to improve professionals’ work performance by promoting efficiency and productivity, and better flow, availability and exploitation of information [32, 33]. Moreover, it has been suggested that digitalisation will change work by fostering increased availability, accessibility, acceptability and quality of health care services [34]. Among others, fast knowledge flow, mobility and asynchronous communication enable better efficiency, productivity and knowledge use, whereas information overload, challenges in time management or technological shortcomings can act as obstacles to achieving improved performance [33]. In general, multiple factors can affect how digitalisation eventually impacts the work of health and social care professionals and whether it succeeds at improving performance. It is not just the digitalisation itself, but also professionals’ views and perceptions define the effects and changes in work caused by digitalisation [22].

Although the role of managers is known to be central to supporting the digital work of professionals, and their understanding of the changes taking place in work has been noted to be crucial, little is known about how well managers recognise the effects of digitalisation experienced by professionals. Ensuring a common understanding of the effects would help all concerned pay more attention to the potential harmful effects of digitalisation, so that the opportunities it offers can be exploited in the best possible way [33]. Considering this, the present study applied a qualitative approach and sought to examine how health and social care professionals and managers perceive the effects of digitalisation on the work of professionals. More precisely, the purpose was to gain up-to-date knowledge about the perceived effects of digitalisation on health and social care work, and how congruently managers describe the effects experienced by professionals.

In this study, health and social care digitalisation, referred also as ‘digital transformation’, indicates that healthcare systems and services are in a transition where more services and actions will be digitalised [35]. In practice, this means increased use of information and communication technologies in health and social care products, services and processes. These include the use of electronic patient/client records, laboratory and imaging information systems, electronic referral feedback, electronic prescription, electronic databases and decision support, remote consultation and training, as well as various portals used in patient contacts and contacts between professionals.

## Methods

### Context

This study was conducted in four large health centres located in different hospital districts in northern, eastern, southern and western Finland. The health centres were in the largest cities in the regions and were selected based on their profile as being advanced in the digitalisation of services and practices, and had acted as pilots in the introduction of new digital services (i.e. telemedicine, online appointment bookings, digital messaging, online symptom checkers, digital coaching, decision support systems, and patient-reported medical history).

In Finland, public health care services are divided into primary health care and specialised medical care. Primary health care refers to health centre services provided by municipalities, which include the promotion of the well-being and health of the population and the prevention, diagnosis and treatment of diseases. Municipalities are also responsible for organising social services, which can be organised in connection with health centres. Health centre work is highly multidisciplinary, typically with a pair of nurse-physicians and, if needed, other social and health care professionals participating in the care of clients as a team [36].

Finland is one of the leading countries in digitalisation [37], and this is reflected in the field of health and social care. As in many other countries, the COVID-19 pandemic was a significant push for the increasing provision and use of online services in the social and health sectors in Finland [38]. The development of digital services has been driven by the eHealth and eSocial Strategy, the aim of which is to support the reform of healthcare and social welfare and the role of citizens in maintaining their own well-being by improving information management and increasing digital services [39].

### Study design and participants

This was a qualitative descriptive study utilising semi-structured focus group interviews with health and social care professionals (*n* = 30) and individual interviews with managers (*n* = 21). Consolidated criteria for reporting qualitative research (COREQ) were followed.

The participants were recruited from the four health centres, where contact persons forwarded invitations to professionals and managers to participate in the study. Willing participants were contacted by e-mail and provided detailed information about the study. All those contacted participated in the study.

Professionals participating in focus groups included registered nurses (*n* = 8), public health nurses (*n* = 5), practical nurses (*n* = 7), physicians (n = 4), social workers (*n* = 2), a social counsellor (*n* = 1) and a digital counsellor (*n* = 1). The majority were female (*n* = 27). Their age varied between 26 and 58 (mean = 40 years), their average work experience in the current organisation was nine years, and 12 years for work experience in total.

Of the participating managers, nine worked as frontline managers, eight as middle managers and four as senior managers. Eighteen had a clinical background (12 nurses, six physicians) and three were non-clinicians. Frontline managers and middle managers led clinical units or teams, whereas senior managers were involved in administrative work. Their age varied between 35 and 65 (mean = 50) and the average amount of leadership experience was eight years.

### Data collection

Data was collected from professionals through eight semi-structured focus group interviews between July and December 2020. This method was chosen because it is suitable for providing insights into how people with certain unifying factors experience an issue of interest [40], in this case the effects of digitalisation on work. We conducted two focus groups in each organisation, and each group had four to six participants from different professional groups. Five interviews were conducted in person on site, and in three focus groups we utilised Microsoft Teams due to the worsening COVID-19-pandemic. Two researchers were present in each interview.

From the managers, the data was collected via individual interviews between July and November 2020 by one researcher using Microsoft Teams or audio call. Individual interviews were performed because they were more flexible to schedule and the number of managers in each health centre was relatively small.

The interview guide was designed together with the authors. The questions were open-ended and deliberately broad, as they were not intended to lead respondents but to allow them to bring up issues that were important and perceived as relevant to their lives. In focus groups, professionals were asked how digitalisation and related changes (long and/or short term) have affected their work, how they have experienced changes, whether there have been any challenges, and what increased digitalisation has required of professionals. Managers were asked the same questions, but from the perspective of how they see digitalisation and related changes have affected the work of professionals.

The clarity and relevance of the questions were first pilot tested by interviewing one healthcare professional and one middle manager who worked in a health centre. In addition, after the first focus group, participants and researchers discussed the questions. The questions were found to be understandable and appropriate for the purposes of the study. Data gathered in the first focus group was included in the study. Also, the test interview with one middle manager was included in the study with the consent of the interviewee.

Three researchers (A-MK, JNa and EL) with previous experience about qualitative interview studies and digitalisation of health and social care conducted the interviews. Prior to the start, participants were reminded of the purpose of the study and were also informed about the researchers’ backgrounds in relation to the topic: i.e. they were not involved in the development of digital services. The interviews were audio recorded with the participants’ permission. The focus groups varied from 41 to 79 min, ultimately taking 501 min in total. The managers’ interviews lasted between 25 and 60 min, ultimately taking 850 min in total. The recordings were transcribed verbatim, generating a total of 168 pages of professionals’ data and 232 pages of managers’ data, with line spacing at 1.15 in 11-point Verdana font.

### Data analysis

**Fig. 1:**
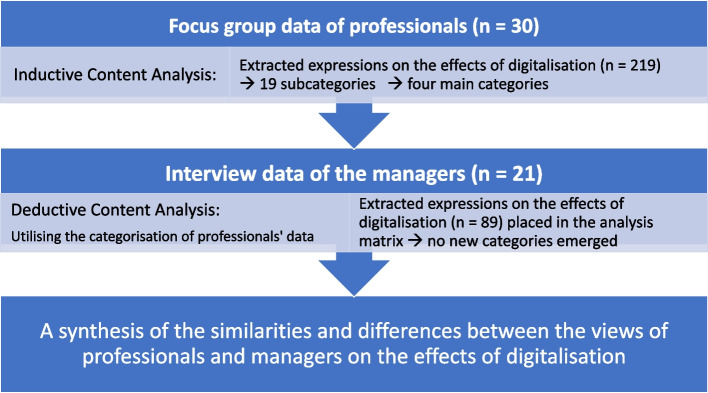
The data analysis process

The data was analysed using qualitative content analysis [41] utilising both inductive and deductive approaches (Fig. 1). Focus groups with professionals were analysed by two researchers (JNä and A-MK) using inductive content analysis, as it allows the research results to emerge from the data without the limitations set by predefined theories or methodologies [42]. This was started by reading through all of the interview transcripts and extracting all the expressions that described the effects of digitalisation on work and renaming them in a simplified but as descriptive a way as possible (*n* = 219). These codes were then grouped based on their content similarity to form subcategories (*n* = 19). As no more new subcategories were formed at the end of the code grouping, this indicated saturation of the data. Finally, the subcategories were merged into four main categories. The logic of the categorisation and the final names of the categories were discussed with the research team until agreement was reached.

A deductive method was chosen for the analysis of managers’ interviews, because it can be used to examine which concepts or phenomena identified by one group (professionals) can be identified from the information collected from another group (managers) [42]. One researcher (EL) analysed the data by utilising the categories created in the analysis of the professionals’ data. The aim was to look at how the issues raised by professionals appear in the views of managers, and through this to look at possible differences and similarities in their views. Despite the use of predefined categorisation, all the expressions describing the effects of digitalisation were extracted from the data because we also wanted to see whether managers highlight any effects that were not raised by professionals. In total, 87 codes were created and placed in the analysis matrix. The analysis of managers’ data did not lead to the creation of additional categories, meaning that all the codes fit into the existing categorisation (i.e. no new effects emerged).

## Results

**Fig. 2:**
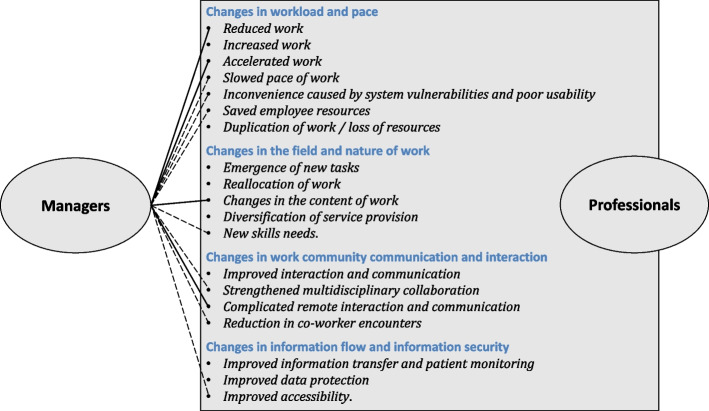
The effects of digitalisation on work raised by professionals and managers ( — perceptions correspond well; --- perceptions somewhat correspond)

The discussions in the different focus groups gave a fairly consistent picture of the effects of digitalisation on the work of professionals, and no group differed significantly from one another. The perceptions of the professionals were separated into four main categories: 1) changes in workload and pace, 2) changes in the field and nature of work, 3) changes in work community communication and interaction, and 4) changes in information flow and information security. There were similarities and differences in the views of professionals and managers. The results are presented comparatively in the sections below and differences are summarised at the end of the results section. Figure 2 illustrates the effects of digitalisation on work brought up by professionals compared to managers.

### Changes in workload and pace

Professionals described the effects of digitalisation on their workload and pace, in terms of *reducing* and *increasing workload*, as well as in terms of *accelerating* and *slowing down work*. In addition, they described the *inconvenience of work caused by information system vulnerabilities and poor usability*, the *saving of employee resources* and the *duplication of work*.

According to professionals, digitalisation has *reduced their work* due to the more active role of the client than before. Clients took more responsibility for their own care by monitoring their own health data and, for example, entering the measured values ​​directly into an electronic system, which used to be the job of a professional. Managers recognised the same phenomenon and added that the client’s pre-completed preliminary information had reduced professionals’ work and sped up reception work. Professionals and managers also agreed that the change from phone calls to digital messaging had decreased the workload, accelerated reaching clients and freed up professionals’ time for other tasks. Moreover, both recognised that advances in technology and robotics have reduced work through electronic drug delivery or decision support systems, for example.

*…digital channel, for example, reduces certain practical work of physicians when some pre-information has already been collected in a structured written form*

*/ Senior manager 2*

*There is no need for the nurse to go to give medicine, as that machine itself relays that now is the time for morning medicine. And then there’s a certain time when it needs to be taken, and then if you don’t, the medication will lock in.*

*/ Professional, focus group 4*

Professionals also pointed out that the amount of *work increased* due to digitalisation, which was hardly recognised by managers at all. According to professionals, new digital practices had increased their overall workload, especially in the early stages of digital service implementations but also in general. Responding to and providing information for clients through messages, directing them to use digital services and providing guidance in their use took time, but it was not always given additional resources. Professionals also stated that messages were not always sufficient for clarifying client matters, but often led to further enquiries, thus adding to their work. In addition, checking for frequently incorrect digital appointment bookings made by clients and redirection of their messages caused significant additional work. Incorrect appointment bookings and the consequent cancellation of receptions and waste of time were the only elements that added work that was brought up by managers.

*[Online appointment booking]…has probably partly made things easier, but then clients may not be able to book the right times, and then they can cancel it from there, and we get idle when a client cancels their online time. / Middle manager 2*

*Yes, they (reception times) are booked for the wrong reasons and people come to the wrong place because they don’t read the instructions. They Come to the nurse’s reception even if the nurse can’t meet that service need. / Professional 2, focus group 8*

As with the workload, the effects of digitalisation on the pace of work were also two-way, meaning that work was perceived to be both *accelerated* and *slowed down* in some respects. Both professionals and managers saw that digitalisation had increased productivity as well as work efficiency and time savings, because remote work and remote appointments had reduced transition times and made it possible to meet more clients/patients in the same or less time than before. Both also brought up that digitalisation had sped up the work by allowing quick consultations between professionals.

*For example, in a digital clinic, the productivity of physicians' work may be ten times higher than, say, traditional reception work. / Senior manager 2*

Themes describing *job slowdown* emerged more in interviews with professionals compared to managers, but some concurring views emerged. Both pointed out that the introduction of new digital services is laborious and requires constant learning and adoption, which slows down the performance of work tasks. Both also identified a paucity of training and guidance, but only professionals emphasised that time allowed for familiarisation is often poor and that learning must be carried out independently alongside work. Moreover, only professionals pointed out that new systems are not always better in terms of functionality than the old ones, but could instead slow down work, as could the search for and processing of data in several existing information systems.

In turn, the hampering effect of the *information system's vulnerabilities and poor usability* was highlighted in interviews with professionals and managers. Difficult-to-use technology was seen as an issue, and different information systems did not always ‘communicate’ with each other, and the systems also sometimes crashed, significantly complicating the work and also exposing professionals to errors in client/patient care.

*We rely on these electronic systems to such an extent… and when the XX system makes significant crashes on a regular basis, then it is very challenging to get this reception work running. In a way, we are no longer prepared to operate as well as in the paper age, that client information would be available, and so on.*

*/ Professional 1, focus group 2*

*It [the information system] doesn't always work. We have days when it didn't work for many hours, and then we don't know who [clients] is coming; we can't document anything. There have been several days like this. / Frontline manager 2*

Digitalisation was also seen to some extent to *save employee resources*. Managers primarily cited automation as reducing the need for human resources, while professionals more widely described that there were more face-to-face appointments available for those clients who needed them the most, because remote services had reduced irreversible appointments and clients did not have to reserve a face-to-face appointment for minor issues, such as to renew a prescription. On the other hand, what the managers didn’t express at all, but the professionals pointed out, was that digitalisation had led to some *duplication of work*. This meant that documentation and internal service requests were now made in several different systems. Moreover, because clients had more than one possible way to contact professionals, they also took advantage of these different channels, with the result being that the same matter could be taken forward by several professionals simultaneously.

*There is a different person in the registration booth, a different person on the phone, and digital services are handled by a different person. There are also two or three nurses for digital services. So, the same thing may then be unknowingly handled by five professionals at worst. If that patient has really been very active.*

*/ Professional 4, focus group 1*

### Changes in the field and nature of work

Professionals described the changes in the field and the nature of their work from the perspectives of the *emergence of new tasks*, *shifting to remote work*, *changes in the work content*, *reallocation of work,* and the *diversification of service provision*, and *new skills needs*.

Professionals stated that digitalisation has led to the *emergence of new tasks*, while managers made little mention of them. The main new task was to act as a digital tutor, as professionals often had to guide both clients and colleagues in the use of digital services. Managers did not mention digital guidance as being important for all professionals, but brought up the formation of the roles of digital nurses and coordinators, who were designated as being responsible for providing digital support. Other new tasks brought to light by the professionals included assessing the conditions and suitability of clients for the use of digital services, and recommending and advertising digital services to clients.

*This remote reception can only be recommended for some clients. Then you take a closer look at how a person can use it, even if it doesn't require many clicks, but you always have to discuss how much it makes them nervous and it can't be recommended to everyone. / Professional 2, focus group 7*

Both professionals and managers brought up *changes in the professionals’ work content* and described it as diversified and expanded when work could also be done digitally. Professionals were able to carry out assessments of the client's need for care remotely using a video call, or monitor their medication digitally, for example. Managers pointed out that patient information systems have brought ‘handy’ operating models to professionals to structure their own work, but this did not emerge in interviews with professionals. Instead, the professionals noted that information systems have led to *reallocation of their work* and referred to a decrease in work performed close to clients/patients, which had been replaced by technical and information system work. This was not seen as a desirable outcome.

*At least, I feel like the work of practical nurses has gone into running these digital things, just these feedback systems and the home measurement results sent by patients. The work of practical nurses has changed a lot. Before, we assisted doctors more, and had some of our own treatment procedures. / Professional 1, focus group 2*

Overall, both professionals and managers noted that teleworking, e-learning, teleconferencing and meeting clients and co-workers remotely had significantly replaced face-to-face meetings, and these changes had grown exponentially, especially due to the COVID-19 pandemic.

Professionals and managers shared the view that digitalisation had created *new skills needs* and continues to demand a lot of learning and adopting. The main new skill need that both groups highlighted was general technical skills in the use of information systems, programs and platforms. Unlike managers, professionals also added that they needed the skills to instruct others in the use. Additionally, both described that professionals need new skills to be able to assess the client’s suitability for digital services, and to communicate and interact with clients remotely. However, there were some differences in perspectives. Where managers described suitability assessment primarily from the perspective of whether the client’s issue required a face-to-face encounter or whether a phone call would be sufficient, professionals more broadly described the skills required to assess the client’s overall situation and access to digital services. Managers noted that professionals must be able to communicate information clearly to clients remotely, but professionals considered their own ability to interact successfully with clients more widely in a digital environment and emphasised that they need to advance their skills relevant to the creation of a sense of connection and presence with clients remotely.

*I must learn, for example, how to make contact with another person online, make clearer gestures, expressions, eye contact. I've been thinking about these types of things myself. / Professional 4, focus group 3*

### Changes in work community communication and interaction

Professionals described that digitalisation has induced changes in how people communicate and interact in work environments. The changes were referred to as an *improvement in interaction and communication* within the work community and as *strengthening of multidisciplinary collaboration*. On the contrary, *difficulties in remote interaction and communication* and a *reduction in co-worker encounters* were also described.

According to the professionals, *interaction and communication within the work community improved*, as digital contacts had made it quick and easy to reach and consult colleagues, and also did not interrupt work in the same way as answering a phone call or a face-to-face conversation. Professionals described digitalisation as *strengthening multidisciplinary collaboration*, as the threshold for contacting colleagues of different professional groups was lower. Collaboration was facilitated by the possibility to contact and send a consultation request to different professionals at the same time. Additionally, professionals emphasised that digitalisation had led to increased collaboration outside their own organisation and that networking with various parties had intensified. One of the managers noted that remote connections may have increased multidisciplinarity, but otherwise the topic was not brought up in managers’ interviews.

*In particular, COVID introduced a mandatory digital leap, which intensified network cooperation. It felt really natural to be connected a lot through digital channels, and then everything started to work through it. A lot of good things have come. /Professional 3, focus group2*

On the other hand, professionals relayed that *interaction had become more difficult* in some respects. Digital communication was described as scarcer than face-to-face, and professionals felt that joint reflection about client cases and discussions about non-work issues had decreased. In addition, many channels for communication posed challenges, as there could be uncertainty about which channel to use. Managers shared the professionals’ views on the rigidity of digital communication, but additionally stated that it was difficult for some professionals to make their voices heard at remote meetings, and that not all things may be clear to everyone following them.

Finally, professionals and managers pointed out that remote meetings and consultations had led to a *reduction in co-worker encounters*. Co-workers were no longer encountered in the same way and as much as before, and the work had become more solitary. These changes were considered negative among professionals, as they missed face-to-face encounters and work methods. Moreover, the decrease in contacts, combined with the frustration associated with the increase in digital workloads, was described to have negatively affected the general atmosphere and cohesiveness of the work community. This was not mentioned by the managers.

*I don’t know if the relationship between nurses has strained a little when we have to do so much of all the extra, or that kind of non-nursing work.*

*/ Professional 1, focus group 2*

*But yes, I still feel that shared coffee moments, food breaks, things like that, they are really important in the life of that work community, and if everything is solely digital now, then we will lose a lot. / Frontline manager 5*

### Changes in information flow and information security

The final topic brought up by the professionals was changes that digitalisation had induced in information flow and security, and this was discussed from the perspectives of *improved information transfer and patient/client monitoring*, *improved data protection* and *improved accessibility*.

Professionals described that common data archives and databases have significantly improved and accelerated the *transfer of information* between different units and service providers, significantly facilitating the work. Monitoring of patient care had also been enhanced considerably thanks to real-time data transfer. Additionally, professionals mentioned how digitisation of referrals had made it easier to handle client matters and reduced the chances of errors that could slow down access to care.

Professionals also saw that digitalisation had *improved data protection* and increased security, as the possibility of errors was lower when client identities or treatment-related issues were not handwritten. Data security had also improved in that viewing client information was traceable, and because digital messages were more likely to go to the right place and the right person than a paper letter.

*In terms of safety, client information is sent and goes to the right person […] and you no longer have to enter clients’ personal data by hand in a sample tube, for example. It has reduced the number of errors. / Professional 2, focus group 5*

Lastly, the professionals described *improved accessibility,* and this was reflected both in the fact that with digital opportunities, professionals reached clients better than before, and clients reached professionals more easily. Better accessibility and its benefits to clients was also brought up by managers, but otherwise they did not describe the effects of digitalisation on information transfer or security-related themes.

In summary, it can be stated that, firstly, managers did not mention any increase in the work of professionals or the unintentional duplication of work that professionals noted as a waste of employee resources. Secondly, professionals described digitalisation as the creation of new general work tasks on top of existing ones, such as acting as a digital tutor for clients and colleagues, which managers saw as belonging mainly to certain designated professionals. Then, significant new competency requirements described by professionals, which were related to guiding others in the use of digital services as well as successful remote interaction with clients, did not appear in the managers’ responses. Nor did they mention the recurring problem of too little time being allocated for professionals to learn and integrate new digital systems and services into work, or that information technology-related work reduced and replaced direct customer contact. Finally, the positive effects of digitalisation on improved multidisciplinary work, better information flow and data security were brought up by professionals but not by managers.

## Discussion

The purpose of this study was to examine and compare how both health and social care professionals and managers describe the effects of digitalisation on the work of professionals. More specifically, we aimed to find out whether managers recognise the potential effects brought up by professionals, and whether similarities and differences exist in their perceptions. Previous research on these micro-level effects of digitalisation is scarce, and to our knowledge, this is the first study comparing the views of professionals and managers. The results showed that professionals and managers share certain perceptions on the effects of digitalisation. However, we also found considerable differences in their responses, meaning that many of the effects described by professionals did not emerge in the interviews with managers.

The results of this study illustrate that changes at work due to digitalisation are often two-sided: on the one hand, it helps to achieve something, but on the other hand it complicates or slows something else down, which supports earlier findings [32, 43]. However, our results suggest that the two-sided effects may be more evident to professionals than for managers. While both groups expressed that digitalisation had reduced workload and facilitated and accelerated the performance of some tasks and functions, professionals also pointed out that it had simultaneously increased workload and slowed down work due to poor usability of the systems or continuous learning required to implement new digital services, for example. The perceptions of the professionals are in line with an earlier study conducted in the homecare environment, stating that digitalisation can have positive effects on better work organisation and time management, for example, but it can also increase workload and pace, and reduce work content management [43]. Other two-way effects of digitalisation have also been identified, such as how it may increase employee autonomy, job satisfaction and a positive image of work, which promote efficiency, but on the other hand, it also increases job monitoring and supervision, which can cause stress and impair employee well-being [13]. It has been stated that constant implementations of new digital services may push managers towards a more authoritarian management style, which employees can experience as bullying or dictatorial [44]. The fact that these problems did not arise in this study may indicate that there were no major problems experienced with digitalisation-related management practices.

Previous research has suggested a possible link between digitalisation and increased inter-professional work [45]. In this study, only the professionals described digitalisation as facilitating consultation and lowering the threshold for communication between professional groups. However, our results also showed that digitalisation had led to a reduction in peer encounters, joint discussions and reflections, which was a negative consequence in the views of both professionals and managers. The results highlight the importance of promoting daily face-to-face contacts in the workplace, as it has also previously been shown that virtualised (distributed) teamwork, with typically less spontaneous discussions, may lead to negative outcomes, such as interpersonal conflicts, higher stress, and less work-related resources among professionals [14]. It is noteworthy that in this study, professionals also felt that the ongoing digital changes and the associated frustration had worsened the atmosphere in teams. Few earlier studies exist on the effects of digitalisation on team climate or social aspects of inter-professional collaboration in the health and social care sector [46]. However, research conducted in other contexts than health and social care suggests that digitalised work and coping with technology-induced changes may indeed trigger conflicts at the workplace [44]. Managers must be alert to combating such phenomena and to anticipating and addressing potential conflict situations to prevent them from escalating.

It is well known that health and social care professionals are continuously required to develop and acquire new knowledge, skills and attitudes about digital technology and digitalising work and services [5, 47]. These requirements may increase stress and impair well-being, especially if the usability of the systems is poor, and professionals experience time pressure or do not have the necessary competence and sufficient time or opportunities to acquire the required competence [48–50]. It is worrying that in this study, managers did little to highlight the new skills needs of professionals other than learning the technical skills of new systems. Nor did they mention the key problem expressed by professionals in combining continuous learning with busy work schedules without sufficient time allocated to familiarisation. Thus, it seems that managers may not fully recognise or see the negative aspects that increased digitalisation brings to professionals. This may lead to perceptions of injustice among professionals which, in turn, has been associated with lower productivity and higher levels of retirement intentions among health care employees, for example [51, 52].

According to multiple studies, a high level of support for using digital tools and leadership that promotes well-being seems to be the best combination to help employees cope with the use of new technologies as well as minimising stress [14, 53, 54]. Thus, to promote the well-being of professionals and to provide them with appropriate support in increasingly digitalised work, identifying staff skills needs and listening to their training aspirations is extremely important in leadership work. According to our findings in addition to technical skills, more attention should be paid to professionals’: 1) ability to communicate and create a sense of connection and presence with clients and colleagues digitally; 2) ability to assess the individual suitability of clients and their situation for digital services; and 3) ability to take on the role of digital tutor, which also seems to be increasingly required of them. These competence needs have also been identified as part of the twelve key digitalisation competencies in the Finnish health and social care sector [55]. In addition to adequate training, the challenges identified in this study could be at least partly prevented by jointly planning how work tasks are performed with new digital technology and what the roles of the different actors are.

Overall, this study showed that the perceptions of managers seem to focus more on the positive effects of digitalisation compared to health and social care professionals. This may be because when implementing new digital health solutions, managers are expected to be advocates of digital health solutions and to demonstrate a visible commitment to the implementation process [56]. In addition, earlier research has suggested that managers need to convince unwilling users to view digitalisation more positively [57]. However, because of these more positive views, managers might be eager to implement more digital solutions that do not support professionals’ work, but rather cause them distress. This highlights the need for managers to be aware of how professionals experience digital technologies and their impact on work. If digital technologies do not support the work of professionals, managers must be ready to abandon non-functional solutions [58]. Thus, as an implication we would recommend that managers listen to end-users’ experiences of digital technologies in order to understand the benefits and effectiveness of digital solutions. In cases where there is no effectiveness or benefits to professionals’ work, managers should estimate whether digital technology is needed.

### Limitations

Certain limitations should be considered when assessing the credibility and transferability of the findings. First, the study was only conducted in a health centre context, in which case the transferability of the results to other contexts must be viewed with caution. On the other hand, the diversity of health centres in the provision of different health and social care services, as well as the strong profiling of participating organisations as active users of digitalisation, increase the likelihood that this study produced usable information that can be utilised in environments that are in the earlier stages of developing and implementing new digital services.

The possible selection of managers and professionals enrolled in the study must also be considered. It is possible that participants were more active, motivated or critical than average in matters related to digitalisation; thus their perceptions about its effects on work may have differed, in either direction, from others. On the other hand, the set of interviews representing different professional groups, different work units and different career stages provided rich data and was a strength of this study. However, this broad approach also has its weaknesses, as our study is not able to provide more in-depth information on the perceived effects of digitalisation according to different professions or types of digital work.

It should be noted that while professionals highlighted some effects of digitalisation on work that managers did not, it does not mean that managers do not agree with the issues raised by professionals. For example, it is possible that managers did not raise themes related to information transfer or security because they may perceive them as obvious. They may have also brought up more issues that are closer to their own work, such as in relation to how much time and resources professionals spend on performing work tasks. The open-ended questions allowed professionals and managers to freely express the effects they felt were most important, and based on the differences in responses, managers apparently did not see all the effects expressed by professionals equally. We did not take the opportunity to ask participants for feedback on the categorisation of the data or the interpretation of the results, which would have increased the credibility of the findings. However, the interviews and the analysis were carried out in close collaboration with several researchers, and repeated discussions were held with the research team at different stages of the study, which increases the trustworthiness of the results. Moreover, the categorisation of data showed saturation, which indicates a sufficient sample size and increases the credibility of the analysis. Finally, it is possible that the shift from face-to-face focus groups to Microsoft Teams may have affected data quality, as the virtual environment hampered the natural rhythm of the conversation and the moderator had to intervene to prevent overlapping and activate silent participants.

## Conclusions

This study provided new insights into the perceived effects of digitalisation on the work of health and social care professionals by comparing the views of professionals and managers. Although their perceptions were partly consistent, the findings suggest that some of the effects of digitalisation and changes in the workplace may receive little or no recognition from managers. They described the effects more positively, albeit also more narrowly, than the professionals did. Managers' positive attitudes about the effects and benefits may be useful when implementing new digital services, but the downside is the risk that the potential negative effects of digitalisation may not be adequately addressed and that managers will adopt systems that do not support the work of professionals.

Increased workload, slowing down of work, new skills requirements, and insufficient time to become acquainted with new systems, among others, can be serious issues if not adequately considered in the implementation of new digital services and systems. Our findings suggest that there is a need for joint discussions on the effects of digitalisation between professionals and different management levels to increase the likelihood that digitalisation will deliver its intended benefits. The results of this study can be utilised as a basis for such discussions, to provide adequate support to professionals, to redesign work and services, and overall to help maximise the benefits and minimise the potential disadvantages of digitalisation for work. To obtain even more detailed information on the effects and possible consequences of digitalisation, it would be useful to carry out further research via occupational group study and in different work environments, and by using quantitative methods. The multifaceted effects of digitalisation not only contribute to the well-being of professionals but are also likely to have a positive impact on the quality of health and social services.

## Data Availability

The datasets generated and analysed during the current study are not publicly available in order to protect of the anonymity of the participants, but they are available from the corresponding author on reasonable request.

## Abbreviations

COVID-19: Coronavirus disease 2019

## References

[1] Keesara S, Jonas A, Schulman K (2020). Covid-19 and health care’s digital revolution. N Engl J Med.

[2] Xie B, Charness N, Fingerman K, Kaye J, Kim MT, Khurshid A (2020). When going digital becomes a necessity: ensuring older adults’ needs for information, services, and social inclusion during COVID-19. J Aging Soc Policy.

[3] Granström E, Wannheden C, Brommels M, Hvitfeldt H, Nyström ME (2020). Digital tools as promoters for person-centered care practices in chronic care? Healthcare professionals’ experiences from rheumatology care. BMC Health Serv Res.

[4] López Peláez A, Marcuello-Servós C. e-Social work and digital society: reconceptualizing approaches, practices and technologies. e-Social work and digital society: re-conceptualizing approaches, practices and technologies 2018;21(6):801–3.

[5] European health parliament. Digital skills for health professionals. Committee on digital skills for health professionals 2016. https://www.healthparliament.eu/wp-content/uploads/2017/09/Digital-skills-for-health-professionals.pdf. Assessed 13 Nov 2022.

[6] OECD. Health in the 21st Century. Putting Data to Work for Stronger Health Systems. OECD Health Policy Studies 2019. https://www.oecd-ilibrary.org/docserver/e3b23f8e-en.pdf?expires=1687857937&id=id&accname=oid013683&checksum=4229E7AF40DC640D31282B8D00DBE6ED. Assessed 12 Nov 2022.

[7] Buntin MB, Burke MF, Hoaglin MC, Blumenthal D (2011). The benefits of health information technology: a review of the recent literature shows predominantly positive results. Health Aff.

[8] Pita-Barros P, Bourek A, Brouwer W, Lehtonen L. Assessing the impact of digital transformation of health services. Report of the EXPH (Expert Panel on effective ways of investing in Health). 2019. https://health.ec.europa.eu/system/files/2019-11/022_digitaltransformation_en_0.pdf. Assessed 1 Nov 2022.

[9] Ross J, Stevenson F, Lau R, Murray E (2016). Factors that influence the implementation of e-health: a systematic review of systematic reviews (an update). Implement Sci.

[10] Nadav J, Kaihlanen A, Kujala S, Laukka E, Hilama P, Koivisto J (2021). How to implement digital services in a way that they integrate into routine work: qualitative interview study among health and social care professionals. J Med Internet Res.

[11] Papoutsi C, Wherton J, Shaw S, Morrison C, Greenhalgh T (2021). Putting the social back into sociotechnical: case studies of co-design in digital health. J Am Med Inform Assoc.

[12] Shaw J, Agarwal P, Desveaux L, Palma DC, Stamenova V, Jamieson T (2018). Beyond “implementation”: digital health innovation and service design. NPJ Digital Med.

[13] Cijan A, Jenič L, Lamovšek A, Stemberger J (2019). How digitalization changes the workplace. Dynamic Relation Manag J.

[14] Bregenzer A, Jimenez P (2021). Risk factors and leadership in a digitalized working world and their effects on employees’ stress and resources: web-based questionnaire study. J Med Internet Res.

[15] Collins S, Yen P, Phillips A, Kennedy MK (2017). Nursing informatics competency assessment for the nurse leader: the delphi study. JONA.

[16] Laukka E, Huhtakangas M, Heponiemi T, Kanste O (2020). Identifying the roles of healthcare leaders in hit implementation: a scoping review of the quantitative and qualitative evidence. Int J Environ Res Public Health.

[17] Hall A, Wilson CB, Stanmore E, Todd C (2017). Implementing monitoring technologies in care homes for people with dementia: a qualitative exploration using normalization process theory. Int J Nurs Stud.

[18] Frennert S (2019). Lost in digitalization? Municipality employment of welfare technologies. disability and rehabilitation. Assist Technol.

[19] Delpha D (2014). Nurse leaders guide to a large-scale information technology implementation. Nurse Lead.

[20] Harteis C, Goller M, Caruso C. Conceptual change in the face of digitalization: challenges for workplaces and workplace learning. Front Educ. 2020;5(1):1–10.

[21] Ferrari A. DIGCOMP: A Framework for Developing and Understanding Digital Competence in Europe. (report EUR 26035), Publications Office of the European Union, Luxembourg. 2013. https://publications.jrc.ec.europa.eu/repository/handle/JRC83167. Assessed 10 Nov 2022.

[22] Meske C, Junglas I (2020). Investigating the elicitation of employees’ support towards digital workplace transformation. Beh Inform Technol.

[23] Evangelista R, Guerrieri P, Meliciani V (2014). The economic impact of digital technologies in Europe. Econ Innov New Technol.

[24] Habibi F, Zabardast MA (2020). Digitalization, education and economic growth: a comparative analysis of Middle East and OECD countries. Technol Soc.

[25] Eldh AC, Sverker A, Bendtsen P, Nilsson E (2020). Health care professionals’ experience of a digital tool for patient exchange, anamnesis, and triage in primary care: qualitative study. JMIR Hum Factors.

[26] Tomasella F, Morgan HM (2021). “Sometimes I don’t have a pulse… and I’m still alive!” Interviews with healthcare professionals to explore their experiences of and views on population-based digital health technologies. Digital Health.

[27] Fagerlund AJ, Holm IM, Zanaboni P (2019). General practitioners’ perceptions towards the use of digital health services for citizens in primary care: a qualitative interview study. BMJ Open.

[28] Lin H (2017). Nurses' satisfaction with using nursing information systems from technology acceptance model and information systems success model perspectives: a reductionist approach. CIN.

[29] Staggers N, Elias BL, Makar E, Alexander GL (2018). The imperative of solving nurses’ usability problems with health information technology. JONA.

[30] Davis FD (1989). Perceived usefulness, perceived ease of use, and user acceptance of information technology. MIS Quarterly.

[31] Rouidi M, AbdElmajid E, Hamdoune A, Choujtani K, Chati A (2022). TAM-UTAUT and the acceptance of remote healthcare technologies by healthcare professionals: a systematic review. Informat Med Unlocked.

[32] Digitalization Changing Work: Employees’ view on the benefits and hindrances. International Conference on Information Technology & Systems: Springer; 2019.

[33] Vuori V, Helander N, Okkonen J (2019). Digitalization in knowledge work: the dream of enhanced performance. Cogn Technol Work.

[34] Lapão LV (2018). Digitalization of healthcare: where is the evidence of the impact on healthcare workforce'performance? Building continents of knowledge in oceans of data: the future of co-created ehealth. IOS Press.

[35] European Commission Expert Panel. Assessing the impact of digital transformation of health services. Expert Panel on Effective Ways of Investing in Health. 2021.

[36] Keskimäki I, Tynkkynen L, Reissell E, Koivusalo M, Syrjä V, Vuorenkoski L (2019). Finland: health system review. Health Syst Transit.

[37] European commission. Directorate-general for communications networks, content and technology. International digital economy and society index 2020 – final report, publications office. 2020. https://data.europa.eu/doi/10.2759/757411.

[38] Rissanen P, Parhiala K, Kestilä L, Härmä V, Honkatukia J, Jormanainen V. Effects of COVID-19 epidemic on the population's service needs, the service system and the economy - rapid impact assessment. Finnish Institute for Health and Welfare (THL). 2020. Report 8/2020. https://www.julkari.fi/bitstream/handle/10024/139694/URN_ISBN_978-952-343-496-7.pdf?sequence=1.

[39] Ministry of Social Affairs and Health. Information to support well-being and service renewal. eHealth and eSocial Strategy 2020. Edita Prima, Helsinki. 2015; Available at: https://julkaisut.valtioneuvosto.fi/bitstream/handle/10024/74459/URN_ISBN_978-952-00-3575-4.pdf?sequence=1&isAllowed=y. Accessed 21.1., 2022.

[40] Parker A, Tritter J (2006). Focus group method and methodology: current practice and recent debate. Int J Res Method Educ.

[41] Elo S, Kyngäs H (2008). The qualitative content analysis process. J Adv Nurs.

[42] Kyngäs H, Mikkonen K, Kääriäinen M. The application of content analysis in nursing science research. : Springer; 2019.

[43] Peña-Casas R, Coster S. The impact of digitalization on job quality in European public services. The case of homecare and employment service workers. 2018.

[44] Melzer SM, Diewald M (2020). How individual involvement with digitalized work and digitalization at the workplace level impacts supervisory and coworker bullying in german workplaces. Soc Sci.

[45] Chao C (2016). The impact of electronic health records on collaborative work routines: a narrative network analysis. Int J Med Inf.

[46] Beckmann M, Dittmer K, Jaschke J, Karbach U, Köberlein-Neu J, Nocon M (2021). Electronic patient record and its effects on social aspects of interprofessional collaboration and clinical workflows in hospitals (eCoCo): a mixed methods study protocol. BMC Health Serv Res.

[47] Konttila J, Siira H, Kyngäs H, Lahtinen M, Elo S, Kääriäinen M (2019). Healthcare professionals’ competence in digitalisation: a systematic review. J Clin Nurs.

[48] Heponiemi T, Kujala S, Vainiomäki S, Vehko T, Lääveri T, Vänskä J (2019). Usability factors associated with physicians’ distress and information system-related stress: cross-sectional survey. JMIR Med Inform.

[49] Kaihlanen A, Gluschkoff K, Laukka E, Heponiemi T (2021). The information system stress, informatics competence and well-being of newly graduated and experienced nurses: a cross-sectional study. BMC Health Serv Res.

[50] Virone C, Kremer L, Breil B (2021). which factors of digitisation bias the work-related stress of healthcare employees? A systematic review. Stud Health Technol Inform.

[51] Heponiemi T, Elovainio M, Laine J, Pekkarinen L, Eccles M, Noro A (2007). Productivity and employees' organizational justice perceptions in long-term care for the elderly. Res Nurs Health.

[52] Heponiemi T, Kouvonen A, Vänskä J, Halila H, Sinervo T, Kivimäki M (2008). Health, psychosocial factors and retirement intentions among finnish physicians. Occup Med.

[53] Salanova M, Llorens S, Cifre E (2013). The dark side of technologies: Technostress among users of information and communication technologies. Int J Psychol.

[54] Knani M, Fournier P, Biron C (2018). Psychosocial risks, burnout and intention to quit following the introduction of new software at work. Work.

[55] Värri A, Tiainen M, Rajalahti E, Kinnunen U, Saarni L, Ahonen O (2020). The definition of informatics competencies in finnish healthcare and social welfare education. Stud Health Technol Inform.

[56] Poon EG, Blumenthal D, Jaggi T, Honour MM, Bates DW, Kaushal R (2004). Overcoming barriers to adopting and implementing computerized physician order entry systems in US hospitals. Health Aff.

[57] Dugstad J, Eide T, Nilsen ER, Eide H (2019). Towards successful digital transformation through co-creation: a longitudinal study of a four-year implementation of digital monitoring technology in residential care for persons with dementia. BMC Health Serv Res.

[58] Laukka E, Pölkki T, Heponiemi T, Kanste O (2022). Finnish primary care leaders’ perceptions of leadership in digital health services: an inductive content analysis. Int J Healthcare Technol Manag.

